# Evaluating the Effectiveness of Cyclin-Dependent Kinase 4/6 Inhibitors in Early- and Very Early-Onset Metastatic Breast Cancer: A Multicenter Study

**DOI:** 10.3390/medicina61010154

**Published:** 2025-01-17

**Authors:** Akif Doğan, Nurullah İlhan, Goncagül Akdağ, Sedat Yıldırım, Mustafa Seyyar, Zeynep Yüksel Yaşar, Hande Nur Erölmez, Heves Sürmeli, Buğra Öztosun, Özlem Nuray Sever, Hatice Odabaş, Mahmut Emre Yıldırım, Devrim Çabuk, Nedim Turan, Mahmut Gümüş

**Affiliations:** 1 Department of Medical Oncology, Health Science University, Sancaktepe, Şehit Prof Dr. İlhan Varank Training Research Hospital, Istanbul 34785, Turkey; nurullahilhan07@gmail.com (N.İ.); herolmez@gmail.com (H.N.E.); 2 Department of Medical Oncology, Health Science University, Kartal Dr. Lütfi Kirdar City Hospital, Istanbul 34865, Turkey; akdaggoncagul@gmail.com (G.A.); rezansedat@hotmail.com (S.Y.); dr_zeynepyuksel@hotmail.com (Z.Y.Y.); hevessurmeli@hotmail.com (H.S.); ozlem.sever@hotmail.com (Ö.N.S.); odabashatice@yahoo.com (H.O.); emremahmutyildirim@gmail.com (M.E.Y.); turan.nedim@hotmail.com (N.T.); 3 Department of Medical Oncology, Faculty of Medicine, Kocaeli University, Kocaeli 41001, Turkey; mustafaseyyar27@hotmail.com (M.S.); devrimcabuk@yahoo.com (D.Ç.); 4 Department of Medical Oncology, Faculty of Medicine, Medeniyet University, Prof. Dr. Süleyman Yalçın City Hospital, Istanbul 34700, Turkey; drbugraoztosun@gmail.com (B.Ö.); mahmut.gumus@saglik.gov.tr (M.G.)

**Keywords:** early-onset breast cancer, very early-onset breast cancer, CDK 4/6 inhibitors, premenopausal metastatic breast cancer

## Abstract

*Background and Objectives*: Early-onset breast cancer (EOBC), particularly in patients under 40, presents with distinct biological characteristics and worse survival outcomes compared to late-onset cases. Despite intensive treatments, EOBC patients, especially those with hormone receptor-positive, HER2-negative (HR+/HER2-) subtypes, show poorer prognosis. CDK4/6 inhibitors, combined with endocrine therapy (ET) have become the standard for HR+/HER2- metastatic breast cancer, yet younger patients are underrepresented in clinical trials. This study aims to evaluate the efficacy of ribociclib and palbociclib with ET in HR+/HER2- metastatic breast cancer, addressing the critical gap in understanding treatment outcomes in younger patient populations. *Materials and Methods*: This multicenter, retrospective study evaluated the efficacy and safety of cyclin-dependent kinase 4 and 6 (CDK 4/6) inhibitors, ribociclib, and palbociclib, in combination with endocrine therapy in patients with hormone receptor-positive and human epidermal growth factor receptor 2-negative metastatic breast cancer. *Results*: A total of 198 patients treated between 2019 and 2023 were analyzed for progression-free survival, overall survival, and prognostic factors. Very early-onset breast cancer, which is diagnosed before the age of 35, was identified as an independent prognostic factor for poor progression-free survival. Additional factors associated with poorer outcomes included liver metastasis, progesterone receptor negativity, high tumor grade, and the concurrent use of fulvestrant with CDK4/6 inhibitors. Both ribociclib and palbociclib demonstrated similar efficacy, and dose reductions due to treatment-related adverse events did not compromise therapeutic outcomes. *Conclusions*: This study is the first to focus specifically on the treatment of early-onset breast cancer with CDK4/6 inhibitors, providing critical insights into the unique challenges faced by this patient population. The findings underscore the urgent need for personalized treatment strategies, routine genetic testing, and dedicated clinical trials designed to address the specific needs of these high-risk subgroups. By advancing our understanding of the clinical and molecular landscape of early-onset breast cancer and very early-onset breast cancer, this study lays the groundwork for improving outcomes in these underserved patients through tailored therapeutic approaches.

## 1. Introduction

Age is a significant factor influencing the biology, aggressiveness, and treatment response of breast cancer [1]. In the literature, early-onset breast cancer (EOBC) is typically defined as occurring under the age of 40, while very early-onset breast cancer (VEOBC) refers to cases under the age of 35 [2]. Patients with EOBC and VEOBC often have worse survival outcomes than older patients, despite receiving more intensive treatments [3,4]. Although this situation has been proven in studies to be associated with the higher prevalence of aggressive subtypes in young breast cancer, many studies have shown that the relatively less aggressive hormone receptor-positive, human epidermal growth factor receptor 2-negative (HR+/HER2-) subtype is specifically associated with worse survival in young breast cancer patients [1,4,5].

EOBC exhibits distinct biological characteristics compared to the late-onset disease. Genetic mutations such as BRCA1, BRCA2, and TP53, along with mechanisms like somatic mutations, chromosomal copy number alterations, microRNA regulation, and DNA methylation, are more frequently observed in these patients [6,7]. For instance, HR+/HER2- patients carrying BRCA2 mutations have a worse prognosis compared to sporadic HR+/HER2- cases, and resistance to cyclin-dependent kinase 4/6 (CDK4/6) inhibitors has been reported in this group [7]. However, it has been demonstrated that PARP inhibitors significantly prolong progression-free survival (PFS) in patients with BRCA mutations [7]. Thus, genetic analysis and personalized treatment approaches are crucial for managing EOBC [8].

In the treatment of HR+/HER2- metastatic breast cancer, the standard approach for patients without visceral crisis involves the addition of CDK4/6 inhibitors to endocrine therapy (ET) [9]. Clinical studies have demonstrated that CDK4/6 inhibitors such as ribociclib, palbociclib, and abemaciclib significantly prolong PFS, with ribociclib and abemaciclib also shown to improve overall survival (OS) [10,11,12,13]. However, younger patients, who are more likely to be premenopausal, are often underrepresented even in studies specifically targeting premenopausal populations. Pivotal trials such as MONALEESA-7, RIGHT-Choice, and Young-PEARL have evaluated the efficacy of CDK4/6 inhibitors in premenopausal patients [10,14,15]. Although these studies include subgroup analyses of younger patients, their representation remains limited, reflecting the broader challenge of addressing EOBC in clinical research. This underrepresentation restricts the ability to draw definitive conclusions about the efficacy of CDK4/6 inhibitors in this age group. Further research focusing on younger patients is essential to developing more tailored treatment strategies for EOBC.

In this study, we assessed the efficacy of ribociclib and palbociclib in combination with endocrine therapy (ET) in patients with HR+/HER2- metastatic breast cancer. By emphasizing the impact of age, this research addresses a gap in the literature, where studies on the effectiveness of CDK4/6 inhibitors in younger patients remain limited and insufficient. Although not the first to explore this topic, our study contributes to the growing body of evidence needed to better understand age-related differences in treatment response.

## 2. Materials and Methods

Our retrospective, multicenter study included premenopausal patients diagnosed with HR+/HER2- metastatic breast cancer who received ET—either an aromatase inhibitor or fulvestrant combined with ovarian suppression therapy—with palbociclib or ribociclib at any line of treatment. This study assessed the efficacy of ribociclib and palbociclib with ET in HR+/HER2- metastatic breast cancer. To ensure a homogeneous cohort, all patients were premenopausal. The research highlights age-related effects, addressing gaps in studies on CDK4/6 inhibitors in younger patients. While not the first study on this topic, it adds to the evidence on age differences in treatment response. This approach aimed to create a more homogeneous cohort by excluding postmenopausal patients, thereby enhancing the reliability of the analysis within this specific age group. The study included patients treated at four centers between 2019 and 2023. Patients may have previously received ET and/or chemotherapy at any stage (adjuvant or metastatic). Postmenopausal women and male patients were excluded from the study.

The age limit for EOBC is considered to be 40, while for VEOBC it is 35. Patients were classified based on their age at the time of metastasis. The clinical and histopathological characteristics of the patients at the initiation of CDK 4/6 inhibitor therapy, along with adverse events associated with these treatments, were recorded. Treatment responses were documented according to the RECIST criteria by reviewing imaging studies. Progression-free survival (PFS) was defined as the time from the initiation of CDK 4/6 inhibitors until disease progression or death from any cause, while overall survival (OS) was defined as the time from the initiation of CDK 4/6 inhibitors until death from any cause.

The primary objective of this study was to evaluate the efficacy of CDK 4/6 inhibitors in patients with EOBC and VEOBC, while the secondary objective was to assess the efficacy of CDK 4/6 inhibitors across the entire patient cohort. This dual approach allows for a comprehensive analysis, addressing the unique characteristics of younger patients while providing insights into broader treatment outcomes.

The data analysis was performed using the IBM SPSS Statistics Version 26 software package. Categorical variables, including clinical and demographic characteristics, were analyzed using Chi-square and Fisher’s exact tests, with a significance threshold of *p* < 0.05. Factors affecting progression-free survival were evaluated using the Kaplan–Meier analysis. Survival times were reported with median and 95% confidence intervals (CIs). Factors found to be statistically significant (*p* < 0.005) in the Kaplan–Meier analysis were included in the univariate and multivariate analyses.

Ethical approval for the study was obtained from the Ethics Committee of Sancaktepe, Şehit Prof. Dr. İlhan Varank Training and Research Hospital with the decision numbers GO 11 September 2024 and 278. This multicenter, retrospective study was conducted in accordance with the Declaration of Helsinki of the World Medical Association.

## 3. Results

### 3.1. Baseline Characteristics

A total of 198 patients were included in the study. The median age of the patients at the time of starting CDK 4/6 inhibitor therapy was 43. Ninety-eight patients (49.5%) were diagnosed with de novo metastatic breast cancer, and one hundred patients (50.5%) were diagnosed with recurrent disease. At the time of treatment initiation, 78 patients (39.5%) were classified as ET-sensitive, while 120 patients (60.5%) were classified as resistant. All patients were estrogen receptor (ER)-positive, while 17 patients (8.6%) were found to be progesterone receptor (PR)-negative. In terms of Eastern Cooperative Oncology Group (ECOG) performance status, 171 patients (86.4%) had a score of 0 and 25 patients (12.6%) had a score of 1. At the time of treatment initiation, 100 patients (50.5%) had isolated bone metastasis, while among 122 patients (61.6%) with visceral disease, 45 patients (23.2%) had liver metastasis. Of the CDK 4/6 inhibitors, ribociclib was used by 108 patients (54.5%) and palbociclib was used by 90 patients (45.5%). In combination therapy, 149 patients (75.3%) were treated with an aromatase inhibitor and 47 patients (23.7%) were treated with fulvestrant. Ovarian suppression therapy was administered to all patients. The clinical and histopathological characteristics of the patient group are presented in Table 1.

### 3.2. Comparison of Disease Characteristics by Age Groups

Patients were categorized into groups based on their age. The analysis revealed that neutropenia was significantly more prevalent in patients aged 40 or younger compared to those over 40 (85.3% vs. 70.8%, *p* = 0.024). However, when comparing patients aged 35 or younger to those older than 35, this difference was not statistically significant (84.2% vs. 73.1%, *p* = 0.117). Similarly, ribociclib use was more common in patients aged 40 or younger than in those using palbociclib (58.3% vs. 41.7%, *p* = 0.045), but this difference did not reach significance between the groups aged 35 or younger and those over 35 (60.5% vs. 44.6%, *p* = 0.072). The progression rate was notably higher in patients aged 35 or younger (61.3% vs. 40.7%, *p* = 0.034), yet no significant difference was detected when comparing patients aged 40 or younger with those older than 40 (52.6% vs. 45.2%, *p* = 0.342). No significant differences were found between the groups regarding progesterone receptor (PR) negativity, the presence of liver metastases, ECOG performance status, response rates to endocrine therapy, or the presence of isolated bone metastases (Table 2).

### 3.3. Survival Outcomes and Safety

**Figure 1:**
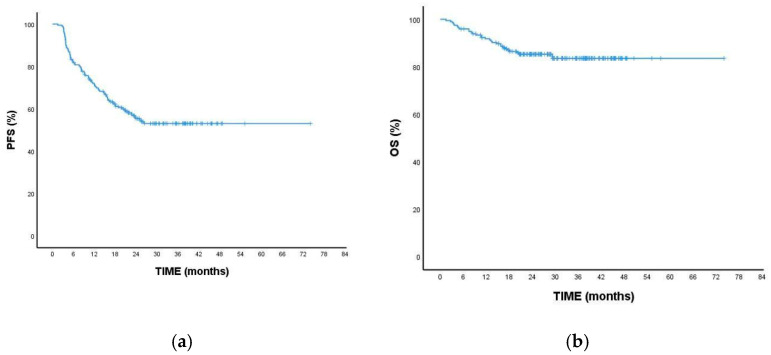
Kaplan–Meier survival analysis curves for all patients: (**a**) progression-free survival and (**b**) overall survival.

The median follow-up duration is 28.8 months (95% CI: 1.5–74.1). During this period, 87 patients experienced disease progression, and 30 patients died. Median PFS and OS could not be reached; the 2-year OS rate was 85.3%, and the PFS rate was 55.4% (Figure 1a,b).

**Figure 2:**
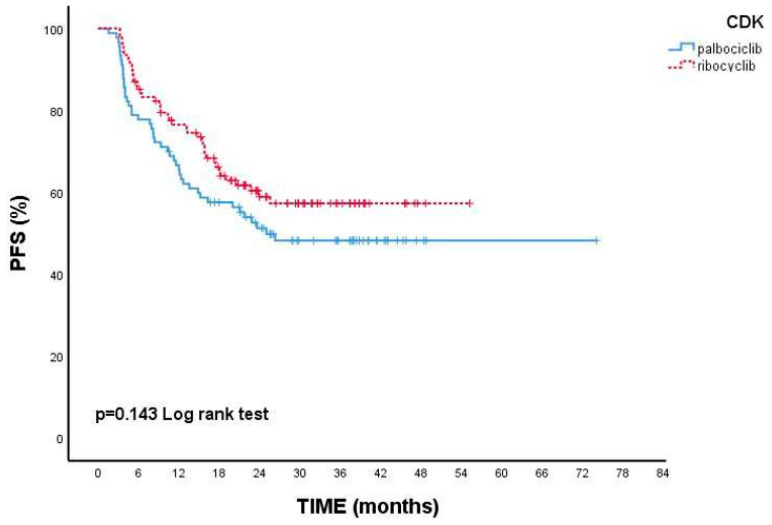
Relationship between CDK4/6 inhibitor types and progression-free survival.

While median PFS could not be reached in patients receiving ribociclib, median PFS was calculated as 25 months in those using palbociclib, but no statistically significant difference was found between the two groups (*p* = 0.143). (Figure 2) In patients with de novo metastatic disease, the 2-year PFS was 60.9%, while in those with recurrent disease, it was 49.9% (*p* = 0.020). In endocrine therapy-sensitive patients, the 2-year PFS was higher at 67.8% (*p* = 0.016). In PR-negative patients, the 2-year PFS was 6.4%, while in PR-positive patients, it was 60.1% (*p* < 0.001). In patients with only bone metastasis, the 2-year PFS was 63%, while in those without bone metastasis, it was 50.6%, and this difference was statistically significant (*p* = 0.016). In patients with liver metastasis, the 2-year PFS was 41.4%, while in those without liver metastasis, it was 63% (*p* = 0.009). In patients with visceral disease, the 2-year PFS was 51.9%, while in those without visceral disease, it was 59%, with no significant difference observed (*p* = 0.212). In patients treated with aromatase inhibitors and CDK 4/6 inhibitors, median PFS could not be reached, while in those treated with fulvestrant, it was calculated as 10.3 months (95% CI: 6.4–14.2) (*p* < 0.001).

**Figure 3:**
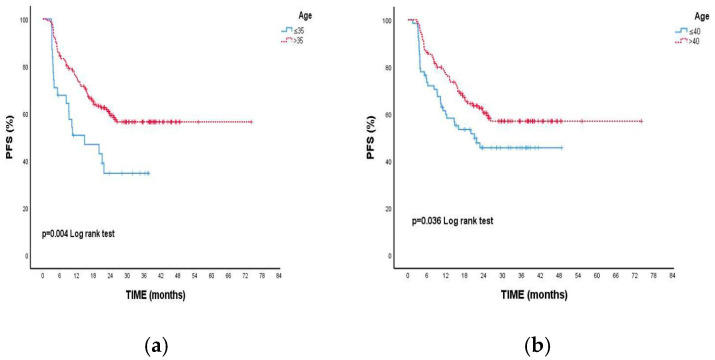
Relationship between age groups and progression-free survival: (**a**) very early-onset breast cancer (VEOBC) and (**b**) early-onset breast cancer (EOBC).

In the group over 40 years of age, the 2-year PFS was 60.5%, while it was 45.8% in those aged 40 and below, with a statistically significant. (95% CI: 21.1 (-)) (*p* = 0.036). (Figure 3a) Similarly, the two-year PFS was 59.3% in patients over 35 years of age, while it was calculated as 34.7% in those under 35, with a statistically significant difference observed. (*p* = 0.004) (95% Cl: 14.8 (1.6–28.1)) (Figure 3b) (Table 3: Comparison of Patient PFS).

Factors that were found to be statistically significant (*p* < 0.05) in the Kaplan–Meier analysis were included in the COX regression analysis.

According to the multivariate COX regression analysis, the following factors were identified as poor prognostic factors: liver metastasis (HR: 2.5, 95% CI: 1.50–4.16, *p* < 0.001), use of fulvestrant with CDK 4/6 inhibitors (HR: 0.36, 95% CI: 0.22–0.57, *p* < 0.001), presence of grade 3 tumors (HR: 4.78, 95% CI: 2.07–11.04, *p* < 0.001), progesterone receptor negativity (HR: 0.41, 95% CI: 0.22–0.77, *p* < 0.001), and being under 35 years of age (HR: 0.39, 95% CI: 0.001).

The multivariate analyses are presented in Table 4.

No new adverse events were observed during the study. Adverse events associated with palbociclib and ribociclib in the EOBC and VEOBC groups are presented in Table 5. Grade 3 or higher adverse events were observed in 125 patients (63.1%), while neutropenia of any grade was noted in 150 patients (75.8%). Dose reduction was required in 39 patients (19.7%), while 159 patients (80.3%) were able to continue treatment at the initial dose. The 2-year PFS was 48.8% in patients receiving the reduced dose and 57.1% in those continuing with the initial dose. However, this difference was not statistically significant (*p* = 0.747).

## 4. Discussion

In our study, we demonstrated that EOBC and VEOBC in HR+/HER2- metastatic breast cancer treated with CDK 4/6 inhibitors in combination with ET is associated with poorer survival, and that very early onset is an independent risk factor for progression.

While young patients are underrepresented in clinical trials, our study addresses this gap by focusing specifically on EOBC and VEOBC [16,17,18]. Our study is the first in the literature to evaluate the efficacy of CDK4/6 inhibitors in metastatic EOBC and VEOBC. In our study, it was found that PFS was significantly lower in patients with metastatic EOBC and VEOBC; the median PFS was 21 months for EOBC and 14.8 months for patients with VEOBC. These findings are consistent with subgroup analyses from the MONALEESA-7, RIGHT-Choice, and Young-PEARL studies, which support the observation of a worse prognosis in young patients [10,14,15]. Our study suggests that the similarity of traditional poor prognostic factors, such as high tumor grade and PR negativity, between patients in the EOBC and VEOBC groups and those with non-early-onset breast cancer indicates the presence of unknown genetic and biological factors related to resistance to CDK 4/6 inhibitors. This highlights the necessity of identifying these factors and developing new targeted therapeutic approaches for this treatment-resistant patient population.

The literature reports that a higher number of genetic anomalies are detected in the young age group, and resistance to CDK4/6 inhibitors can occur [19]. For example, a worse prognosis has been reported in HR+/HER2- patients with BRCA2 mutations, and PARP inhibitors have been shown to significantly extend PFS in these patients [19]. Although the lack of genetic analysis in our study is an important limitation, it emphasizes the need for genetic testing in EOBC. Specifically, as seen in the PADA-1 study, monitoring ESR1 mutations with ctDNA and personalizing treatment may be a promising approach for this patient group [20].

Additionally, our study identified high grade, PR negativity, the presence of liver metastasis, and the concurrent use of fulvestrant with CDK 4/6 inhibitors as factors that increase the risk of progression. This aligns with the existing literature, emphasizing that treatment should be carefully planned and monitored, especially in patients with additional poor prognostic factors [21]. In our study, it was observed that ribociclib was used significantly more frequently in the EOBC group. This can be attributed to the fact that Monaleesa-7, the only study conducted in the premenopausal patient group, was carried out with ribociclib. However, this difference did not reach statistical significance in the VEOBC group, which we believe is primarily due to the limited number of patients in this group. Despite the higher rate of ribociclib use, the efficacy of both drugs was found to be similar in our study. This indicates that the poor prognosis observed in EOBC and VEOBC patients is not dependent on the type of drug used but rather on the biological and genetic factors associated with these diseases [17,21]. Neutropenia was the most common side effect, consistent with pivotal trials [10,11,13]. Dose reductions occurred in 20% of patients due to side effects, with no observed loss in efficacy, reinforcing that dose reduction does not impact CDK4/6 inhibitor effectiveness [21,22,23,24]. The alignment of our findings with the broader literature further affirms the robustness and credibility of our study results.

Our study specifically focuses on HR+/HER2- metastatic EOBC and VEOBC patients treated with ribociclib and palbociclib, representing a larger cohort of this patient group compared to previous studies. It investigates the impact of age on drug efficacy. The retrospective design, the inability to assess abemaciclib, which is not included in the reimbursement scope in our country, and the lack of genetic analyses in the patient cohort are significant limitations of our study, as they prevent a deep examination of the genetic and biological differences in early-onset disease. Given these limitations, it is clear that genetic analyses and results from prospective studies are critical for further improving treatment approaches for this patient group.

## 5. Conclusions

Our study is the first to evaluate the efficacy of ribociclib and palbociclib in EOBC and VEOBC HR+/HER2- metastatic breast cancer patients. We found that PFS is significantly lower in these groups, with VEOBC emerging as an independent prognostic factor for progression. Additional poor prognostic factors identified include high tumor grade, PR negativity, liver metastasis, and the concurrent use of CDK4/6 inhibitors with fulvestrant. Importantly, no significant differences in efficacy were observed between ribociclib and palbociclib, and dose reductions due to side effects did not compromise treatment outcomes. The lack of genetic analyses is a notable limitation of our study, underscoring the critical need for routine genetic testing and personalized treatment strategies for metastatic EOBC. This study highlights the unique challenges of managing HR+/HER2- EOBC and VEOBC, emphasizing the importance of tailored approaches to improve outcomes in this underserved population.

## Figures and Tables

**Table 1:** Data on Sociodemographic and Clinical Characteristics (n = 198).

Variables	N	%
**Age**		
Mean ± SD	41.99 ± 5.82	
Median (min–max)	43.0 (25–51)	
≤40	68	34.3
>40	130	65.7
≤35	31	15.7
>35	167	84.3
**ECOG ^1^ Performance Status**		
0	171	86.4
1	25	12.6
2	2	1.0
**Histologic Type**		
IDC ^2^	178	89.9
Other	20	10.1
**ER ^3^ Percentage**		
1–10	2	1.0
11–40	8	4.0
40–90	126	63.6
>90	62	31.3
**PR ^4^ Percentage**		
1–10	54	27.3
11–40	36	18.2
40–90	85	42.9
>90	23	11.6
**PR**		
Negative	17	8.6
Positive	181	91.4
**Ki67**		
≤14	36	18.2
>14	162	81.8
**Grade**		
1	38	19.2
2	128	64.6
3	32	16.2
**De novo Metastatic**		
No	100	50.5
Yes	98	49.5
**Adjuvant/Neoadjuvant CT ^5^**		
Not received	12	12.0
Received	88	88.0
**Adj HT ^6^**		
Not received	5	5.0
Received	95	95.0
**Liver Metastasis**		
Absent	152	76.8
Present	45	23.2
**Bone only**		
No	122	61.6
Yes	76	38.4
**Visceral/Nonvisceral**		
Nonvisceral	98	49.5
Visceral	100	50.5
**Number of Metastatic Sites**		
1	115	58.1
2	51	25.8
3 or more	32	16.1
**If CT Received, When**		
Not received	66	35.7
Adjuvant	62	33.5
Adjuvant+Metastatic	26	14.1
Metastatic	31	16.7
**Received CT in Stage 4**		
No	140	71.1
Yes	57	28.9
**Received HT in Stage 4**		
No	113	57.7
Yes	83	42.3
**Endocrine Resistance**		
Primary resistant	6	3.0
Secondary resistant	114	57.5
Sensitive	78	39.5
**Concurrent CDK ^7^ Inhibitor Treatment**		
Fulvestrant	47	23.7
Letrozole	149	75.3
Other	2	1.0
**CDK Inhibitor Overall Line of Therapy**		
1	112	56.6
2	77	38.9
3 or more	9	4.5
**Type of CDK Inhibitor**		
Palbociclib	90	45.5
Ribociclib	108	54.5
**Dose Reduction**		
No	159	80.3
Yes	39	19.7
**Grade 3 Toxicity**		
No	125	63.1
Yes	73	36.9
**Neutropenia**		
No	48	24.2
Yes	150	75.8
**Progression**		
No	111	56.1
Yes	87	43.9
**Mortality**		
Alive	168	84.8
Deceased	30	15.2
**Follow-Up Duration (Months)**		
Mean ± SD	28.86 ± 12.82	
Median (min–max)	28.8 (1.5–74.1)	

^1^ Invasive ductal carcinoma; ^2^ Eastern Cooperative Oncology Group; ^3^ estrogen receptor; ^4^ progesterone receptor; ^5^ chemotherapy; ^6^ hormone therapy; ^7^ cyclin-dependent kinase.

**Table 2:** Comparison of Disease Characteristics by Age Groups.

	**Age**					
	≤40	>40		≤35	>35	
Variables	N (%)	N (%)	*p*	N (%)	N (%)	*p*
**ECOG ^1^ Performance Status**						
0	63 (92.6)	108 (83.1)	0.058 ^b^	29 (93.5)	142 (85)	0.320 ^b^
1	4 (5.9)	21 (16.2)		2 (6.5)	23 (13.8)	
2	1 (1.5)	1 (0.8)		0 (0)	2 (1.2)	
**Histologic Type**						
IDC ^2^	65 (95.6)	113 (86.9)	0.055 ^a^	30 (96.8)	148 (88.6)	0.211 ^a^
Other	3 (4.4)	17 (13.1)		1 (3.2)	19 (11.4)	
**ER ^3^ Percentage**						
11–40	0 (0)	8 (6.2)	0.051 ^a^	0 (0)	8 (4.8)	0.343 ^a^
40–90	41 (61.2)	85 (65.9)		19 (61.3)	107 (64.8)	
>90	26 (38.8)	36 (27.9)		12 (38.7)	50 (30.3)	
**PR ^4^ Percentage**						
1–10	22 (32.4)	32 (24.6)	0.443 ^a^	9 (29)	45 (26.9)	0.872 ^a^
11–40	9 (13.2)	27 (20.8)		7 (22.6)	29 (17.4)	
40–90	28 (41.2)	57 (43.8)		12 (38.7)	73 (43.7)	
>90	9 (13.2)	14 (10.8)		3 (9.7)	20 (12)	
**PR**						
Negative	6 (8.8)	11 (8.5)	0.931 ^a^	2 (6.5)	15 (9)	0.748 ^b^
Positive	62 (91.2)	119 (91.5)		29 (93.5)	152 (91)	
**Ki67**						
≤14	7 (10.3)	29 (22.3)	0.067 ^a^	3 (9.7)	33 (19.8)	0.181 ^a^
>14	61 (89.7)	101 (77.7)		28 (90.3)	134 (80.2)	
**Grade**						
1	12 (17.6)	26 (20)	0.874 ^a^	3 (9.7)	35 (21)	0.324 ^a^
2	44 (64.7)	84 (64.6)		23 (74.2)	105 (62.9)	
3	12 (17.6)	20 (15.4)		5 (16.1)	27 (16.2)	
**De Novo Metastatic**						
No	35 (51.5)	65 (50)	0.844 ^a^	15 (48.4)	85 (50.9)	0.797 ^a^
Yes	33 (48.5)	65 (50)		16 (51.6)	82 (49.1)	
**Adjuvant/Neoadjuvant CT ^5^**						
Not received	2 (5.7)	10 (15.4)	0.256 ^b^	1 (6.7)	11 (12.9)	0.687 ^b^
Received	33 (94.3)	55 (84.6)		14 (93.3)	74 (87.1)	
**Adjuvant HT ^6^**						
Not received	2 (5.7)	3 (4.6)	0.810 ^a^	1 (6.7)	4 (4.7)	0.584 ^b^
Received	33 (94.3)	62 (95.4)		14 (93.3)	81 (95.3)	
**Liver Metastasis**						
Absent	53 (77.9)	99 (76.2)	0.777 ^a^	28 (90.3)	124 (74.3)	0.052 ^a^
Present	15 (22.1)	31 (23.8)		3 (9.7)	43 (25.7)	
**Bone only**						
No	39 (57.4)	83 (63.8)	0.372 ^a^	17 (54.8)	105 (62.9)	0.398 ^a^
Yes	29 (42.6)	47 (36.2)		14 (45.2)	62 (37.1)	
**Visceral/Nonvisceral**						
Nonvisceral	37 (54.4)	61 (46.9)	0.317 ^a^	19 (61.3)	79 (47.3)	0.153 ^a^
Visceral	31 (45.6)	69 (53.1)		12 (38.7)	88 (52.7)	
**Number of Metastatic Sites**						
1	46 (67.6)	69 (53.1)	0.139 ^a^	20 (64.5)	95 (56.9)	0.723 ^a^
2	14 (20.6)	37 (28.5)		7 (22.6)	44 (26.3)	
3 or more	8 (11.8)	24 (18.5)		4 (12.9)	28 (16.8)	
**If CT Received, When**						
Not received	26 (39.4)	40 (33.6)	0.200 ^a^	12 (40)	54 (34.8)	0.873 ^a^
Adjuvant	26 (39.4)	36 (30.3)		9 (30)	53 (34.2)	
Adjuvant+Metastatic	7 (10.6)	19 (16)		5 (16.7)	21 (13.5)	
Metastatic	7 (10.6)	24 (20.2)		4 (13.3)	27 (17.4)	
**Received CT in Stage 4**						
No	53 (79.1)	87 (66.9)	0.074 ^a^	21 (70)	119 (71.3)	0.889 ^a^
Yes	14 (20.9)	43 (33.1)		9 (30)	48 (28.7)	
**Received HT in Stage 4**						
No	41 (61.2)	72 (55.8)	0.470 ^a^	17 (56.7)	96 (57.8)	0.905 ^a^
Yes	26 (38.8)	57 (44.2)		13 (43.3)	70 (42.2)	
**Endocrine Resistance**						
Primary resistant	3 (4.4)	3 (2.3)	0.696 ^b^	1 (3.2)	5 (3)	0.945 ^a^
Secondary resistant	39 (57.4)	75 (57.7)		17 (54.8)	97 (58.1)	
Sensitive	26 (38.2)	52 (40)		13 (41.9)	65 (38.9)	
**Concurrent CDK ^7^ Inhibitor Treatment**						
Fulvestrant	18 (26.5)	29 (22.7)	0.552 ^a^	8 (25.8)	39 (23.6)	0.795 ^a^
Letrozole	50 (73.5)	99 (77.3)		23 (74.2)	126 (76.4)	
**CDK inhibitor Overall Line of Therapy**						
1	43 (63.2)	69 (53.1)	0.376 ^a^	19 (61.3)	93 (55.7)	0.821 ^a^
2	22 (32.4)	55 (42.3)		11 (35.5)	66 (39.5)	
3 or more	3 (4.4)	6 (4.6)		1 (3.2)	8 (4.8)	
**Type of CDK Inhibitor**						
Palbociclib	24 (35.3)	66 (50.8)	**0.038 ^a^**	14 (45.2)	76 (45.5)	0.972 ^a^
Ribociclib	44 (64.7)	64 (49.2)		17 (54.8)	91 (54.5)	
**Dose Reduction**						
No	55 (80.9)	104 (80)	0.882 ^a^	24 (77.4)	135 (80.8)	0.606 ^a^
Yes	13 (19.1)	26 (20)		7 (22.6)	32 (19.2)	
**Grade 3 Toxicity**						
No	41 (60.3)	84 (64.6)	0.550 ^a^	19 (61.3)	106 (63.5)	0.617 ^a^
Yes	27 (39.7)	46 (35.4)		12 (38.7)	61 (36.5)	
**Neutropenia**						
No	10 (14.7)	38 (29.2)	**0.024 ^a^**	6 (19.4)	42 (25.1)	0.489 ^a^
Yes	58 (85.3)	92 (70.8)		25 (80.6)	125 (74.9)	
**Progression**						
No	33 (48.5)	78 (60)	0.123 ^a^	12 (38.7)	99 (59.3)	**0.034 ^a^**
Yes	35 (51.5)	52 (40)		19 (61.3)	68 (40.7)	
**Mortality**						
Alive	58 (85.3)	110 (84.6)	0.899 ^a^	26 (83.9)	142 (85)	0.751 ^b^
Deceased	10 (14.7)	20 (15.4)		5 (16.1)	25 (15)	

^a^: Pearson Chi-square test, ^b^: Fisher’s exact test, *p* < 0.05 considered statistically significant. ^1^ Invasive ductal carcinoma; ^2^ Eastern Cooperative Oncology Group; ^3^ estrogen receptor; ^4^ progesterone receptor; ^5^ chemotherapy; ^6^ hormone therapy; ^7^ cyclin-dependent kinase.

**Table 3:** Comparison of Patient PFS (Progression-Free Survival).

PFS (Months)	2-Year (%)	5-Year (%)	Median (%95 CI)	*p*-Value
**Overall**	55.4	53.1	- (-)	
**Age Group 1**				
≤40	45.7	-	21.1 (-)	0.036
>40	60.5	57.0	- (-)	
**Age Group 2**				
≤35	34.7	-	14.8 (1.6–28.1)	0.004
>35	59.3	56.5	- (-)	
**ER ^1^ Percentage**				
11–40	45.0	-	20.5 (8.8–32.1)	0.174
40–90	50.1	48.6	25.0 (-)	
>90	64.7	-	- (-)	
**PR ^2^ Percentage**				
1–10	32.8	-	12.3 (6.18–18.4)	<0.001
11–40	52.3	-	25.0 (-)	
40–90	63.3	61.5	- (-)	
>90	78.3	-	- (-)	
**PR Status**				
Negative	6.4	-	11.1 (5.5–16.6)	<0.001
Positive	60.1	57.5	- (-)	
**Ki67**				
≤14	69.3	69.3	- (-)	0.076
>14	52.1	-	26.3 (-)	
**Grade**				
1	69.9	69.9	- (-)	<0.001
2	58.5	-	- (-)	
3	26.1	-	10.6 (6.6–14.5)	
**De Novo Metastatic**				
No	49.9	-	22.6 (-)	0.020
Yes	60.9	59.1	- (-)	
**Adjuvant/Neoadjuvant CT ^3^**				
No	58.3	-	- (-)	0.289
Yes	48.8	-	22.6 (-)	
**Adjuvant HT ^4^**				
No	40.0	-	15.8 (13.7–17.8)	0.982
Yes	50.5	-	- (-)	
**Liver Metastasis**				
None	59.7	-	- (-)	0.009
Present	41.4	41.4	14.6 (7.1–22.2)	
**Isolated Bone Metastasis**				
No	50.6	46.8	25.0 (-)	0.016
Yes	63.0	-	- (-)	
**Visceral/Non-visceral Metastasis**				
Non-visceral	59.0	-	- (-)	0.212
Visceral	51.9	48.8	26.3 (-)	
**Number of Metastatic Sites**				
1	59.5	-	- (-)	0.249
2	50.2	44.9	25.5 (13.1–38.1)	
3 or more	49.1	-	22.6 (-)	
**CT Timing**				
None	73.2	-	- (-)	<0.001
Adjuvant	58.8	-	- (-)	
Adjuvant + Metastatic	26.9	-	9.2 (-)	
Metastatic	36.4	31.8	18.9 (11.6–26.2)	
**CT While in Stage 4**				
No	66.2	-	- (-)	<0.001
Yes	32.3	30.0	15.5 (12.2–18.8)	
**HT While in Stage 4**				
No	64.0	-	- (-)	0.004
Yes	44.0	-	16.3 (7.1–25.4)	
**Endocrine Resistance**				
Primary	50.0	-	5.7 (-)	0.016
Secondary	47.1	-	21.1 (-)	
Sensitive	67.8	61.1	- (-)	
**Concurrent CDK Inhibitor Treatment ^5^**				
Fulvestrant	21.6	-	10.3 (6.4–14.2)	<0.001
Letrozole	66.5	63.3	- (-)	
**CDK Inhibitor Line of Therapy**				
1st	63.7	59.2	- (-)	0.014
2nd	46.1	-	18.9 (-)	
3rd or later	33.3	-	8.3 (8.1–8.5)	
**CDK Inhibitor**				
Palbociclib	51.2	48.2	25.0 (-)	0.143
Ribociclib	58.9	-	- (-)	
**Dose Reduction**				
No	57.1	54.1	- (-)	0.747
Yes	48.8	-	21.7 (-)	
**Grade 3 Toxicity**				
No	58.1	55.5	- (-)	0.735
Yes	51.5	-	25.5 (-)	
**Neutropenia**				
No	47.0	43.6	16.1 (5.4–26.6)	0.123
Yes	58.1	-	- (-)	

^1^ Estrogen receptor; ^2^ progesterone receptor; ^3^ chemotherapy; ^4^ hormonotherapy; ^5^ cyclin-dependent kinase.

**Table 4:** Multivariate Cox Regression Results for Various Clinical Variables Regarding Progression Risk.

Variables	HR (95% CI)	*p*
**Age Group**		
≤35	ref	
>35	0.39 (0.22–0.68)	**0.001**
**PR ^1^**		
Negative	ref	
Positive	0.41 (0.22–0.77)	**0.006**
**Grade**		**<0.001**
1	ref	
2	1.76 (0.82–3.75)	0.143
3	4.78 (2.07–11.04)	**<0.001**
**Liver Metastasis**		
Absent	ref	
Present	2.50 (1.50–4.16)	**<0.001**
**Simultaneous CDK ^2^ Inhibitor Treatment**		
Fulvestrant	ref	
Letrozole	0.36 (0.22–0.57)	**<0.001**

^1^ Progesterone receptor; ^2^ cyclin-dependent kinase −2 log likelihood = 253.79, *p* < 0.001.

**Table 5:** Hematologic and Non-Hematologic Toxicities by Age Group and Severity Grade.

	≤40		>40	
Variables	Grade 1–2 N (%)	Grade 3–4 N (%)	Grade 1–2 N (%)	Grade 3–4 N (%)
Neutropenia	46 (37.4)	12 (41.4)	77 (34.5)	15 (34.9)
Leukopenia	39 (31.7)	7 (24.1)	67 (30.0)	15 (34.9)
Anemia	21 (17.1)	5 (17.2)	40 (17.9)	8 (18.6)
Thrombocytopenia	12 (9.8)	3 (10.3)	21 (9.4)	1 (2.3)
ALT ^1^ Elevation	3 (2.4)	2 (6.9)	16 (7.2)	2 (4.7)
QTc Prolongation	2 (1.6)	0 (0.0)	2 (0.9)	1 (2.3)
	**≤35**		**>35**	
Neutropenia	20 (38.5)	5 (38.5)	103 (35.0)	22 (37.3)
Leukopenia	15 (28.8)	4 (30.8)	91 (31.0)	18 (30.5)
Anemia	10 (19.2)	3 (23.1)	51 (17.3)	10 (16.9)
Thrombocytopenia	4 (7.7)	1 (7.7)	29 (9.9)	3 (5.1)
ALT Elevation	2 (3.8)	0 (0.0)	17 (5.8)	4 (6.8)
QTc Prolongation	1 (1.9)	0 (0.0)	3 (1.0)	1 (1.7)

^1^ Alanine Aminotransferase.

## Data Availability

The data presented in this study are available on request from the corresponding author. Any requests will be reviewed against compliance with ethical, scientific, regulatory, and legal requirements. Requests to access the datasets should be directed to drakifd@gmail.com.
